# Sirtinol promotes PEPCK1 degradation and inhibits gluconeogenesis by inhibiting deacetylase SIRT2

**DOI:** 10.1038/s41598-017-00035-9

**Published:** 2017-02-28

**Authors:** Mingming Zhang, Yida Pan, Robert G. Dorfman, Yuyao Yin, Qian Zhou, Shan Huang, Jie Liu, Shimin Zhao

**Affiliations:** 1 Department of Gastroenterology, Nanjing Drum Tower Hospital, Medical School of Nanjing University, Nanjing, China; 2 Department of Digestive Diseases, Huashan Hospital, Fudan University, Shanghai, China; 3 Northwestern University Feinberg School of Medicine, Chicago, IL USA; 4 School of Life Sciences, Fudan University, Shanghai, China; 5 Department of Pathology, The Second Hospital of Anhui Medical University, Anhui Medical University, Hefei, China

## Abstract

Phosphoenolpyruvate carboxykinase 1 (PEPCK1) is the critical enzyme for gluconeogenesis and is linked with type II diabetes. Previous studies have found that SIRT2, a deacetylase, plays an important role in deacetylating PEPCK1 and little is known about the anti-diabetic activity of SIRT2 inhibitors. In this study, we investigated the anti-diabetic effects of sirtinol, a SIRT2 inhibitor, on cell gluconeogenesis *in vivo* and *in vitro*. Immunoblotting analysis revealed that sirtinol significantly decreased the protein level of PEPCK1, and was accompanied by the hyperacetylation of PEPCK1 as well as decreased glucose output in a dose-dependent manner. Furthermore, sirtinol exerted little impact on endogenous PEPCK1 levels in *SIRT2*-knockdown cells. The *in vitro* experiments further confirmed the *in vivo* data; sirtinol decreased liver PEPCK1 protein level and prevented pyruvate-induced blood glucose from increasing. Based on our results, the rate-limiting enzyme PEPCK1 is the primary target of sirtinol, and the inhibition of SIRT2 activity may play an important role in cell gluconeogenesis. Thus, SIRT2 may be a novel molecular target for diabetes therapy and may thus shed light on the underlying diabetes treatment mechanisms of sirtinol.

## Introduction

Diabetes is a serious worldwide problem threatening the health of millions of people. It has been demonstrated that oral hypoglycemic agents (OHA) are ineffective means of long-term therapy for Type II diabetic patients, and the involvement of signaling networks of OHA are still not clearly understood^1^. Consequently, there is an urgent need to develop new agents for diabetes therapy, as well as to figure out special targets that can be influenced by drugs.

Post-translational modifications (PTMs) have received widespread attention for being a means of rapid response to changes in cellular metabolic status as well as regulation via upstream signaling. Acetylation, an evolutionarily conserved post-translational modification, has been identified in metabolic enzymes and has played key roles in metabolic regulation^2, 3^. PEPCK1 is an important marker in the evaluation of type II diabetes^4, 5^, and plays an important role in gluconeogenesis by catalyzing the first committed and rate-limiting step mainly in the liver, where it maintains glucose homeostasis^6–9^. Due to the important role of PEPCK1, its regulation has been extensively studied. Both yeast and human PEPCK1 has been found to have acetylation and its catalytic activity is inactivated following this acetylation^3, 10^. Lys70, Lys71, and Lys594 of human PEPCK1 was found to be acetylated, and acetylation of these sites led to decreased protein stability, reduced protein levels, and decreased gluconeogenesis without affecting mRNA levels^3^.

There are four classes (I-IV) of deacetylases; Sirtuins (also known as SIRTs) are NAD^+^-dependent class III HDACs^11^. In mammals, seven SIRT homologues have been identified (SIRT1-7)^12, 13^. SIRT2 has been broadly conserved in evolution from bacteria to mammalian species and catalyzes a wide range of biological processes including gene expression, development, and metabolism. Its enzymatic reaction removes the acetyl group from lysine residues and is accompanied with hydrolysis of NAD to generate nicotinamide (NAM), lysine, and O-acetyl-ADP-ribose. Consequently, NAM can be serve as an inhibitor to this kind of enzymatic reaction^11, 14–16^. SIRT2 is primarily a cytoplasmic protein, and tubulin^17^ as well as PEPCK1^3^ are well known substrates of this deacetylase. As a SIRT2 inhibitor, sirtinol has been shown to have anti-tumor^18–22^ and anti-inflammatory^23, 24^ properties, but its impact on metabolism as well as its molecular mechanisms of action have not yet been reported.

In the present study, we focused on the anti-gluconeogenesis effect of sirtinol and explored its molecular mechanisms. We discovered that sirtinol-induced acetylation plays a critical role in protein post-translational modification of PEPCK1 and cell gluconeogenesis by targeting SIRT2. Additionally, the hypoglycemic effects of sirtinol on glucose output and gluconeogenesis were confirmed *in vitro* as well as *in vivo*.

## Results

### Sirtinol increases the acetylation of PEPCK1 by inhibiting the activity of SIRT2

**Figure 1:**
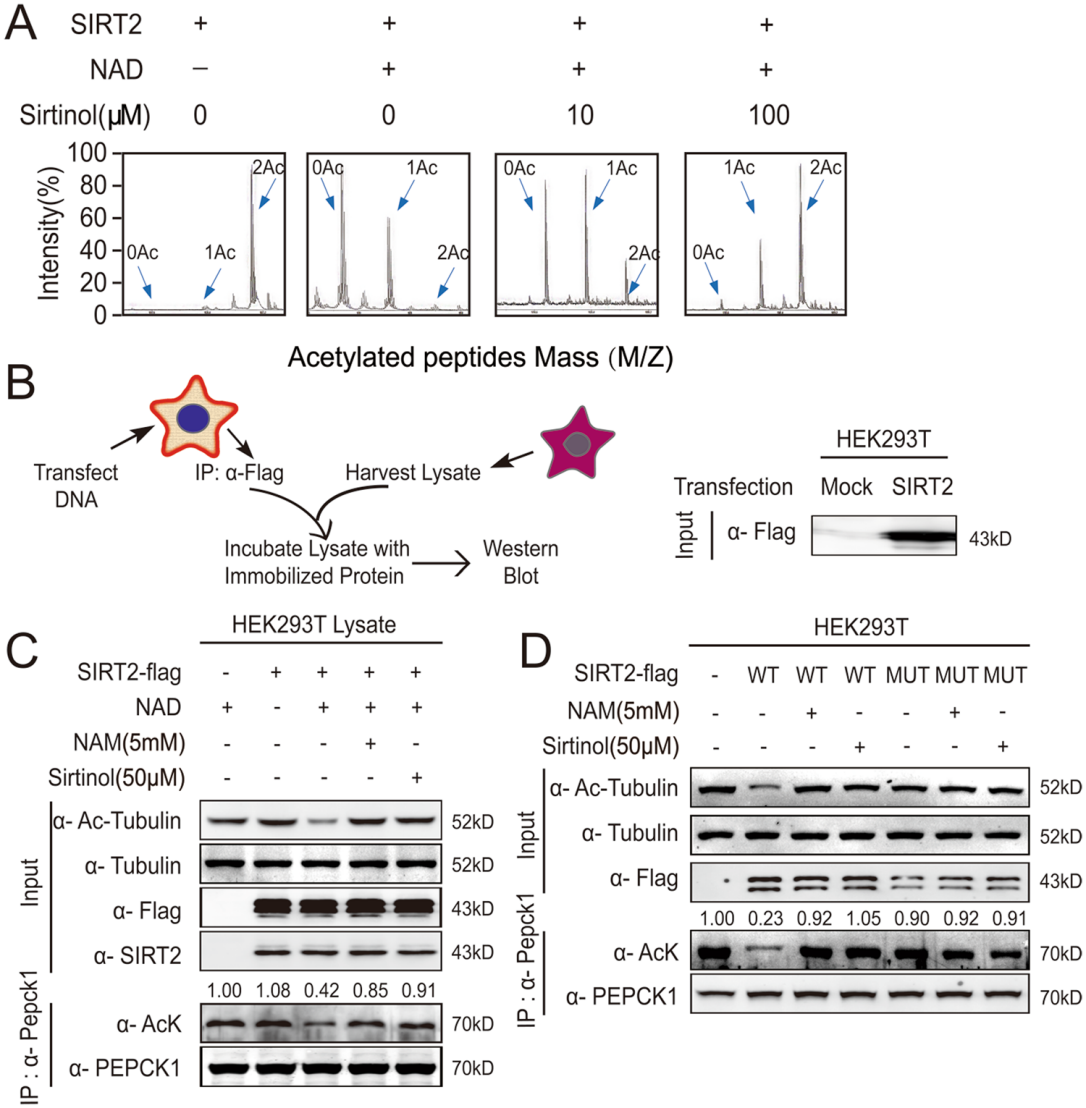
Sirtinol increases the acetylation of PEPCK1 by inhibiting the activity of SIRT2. (**A**) SIRT2 deacetylated toward PEPCK1 (K70, 71) acetylated peptides were assayed in the presence of increasing concentrations of sirtinol, as indicated. The deacetylated peptides were analyzed by MS are shown. (**B**) Schematic diagram of *in vitro* tubulin and PEPCK1 deacetylation assay (left). The visualized expression SIRT2 protein by Western blotting with specific anti-FLAG antibody are shown (right). (**C**) The immunoprecipitated protein corresponding to SIRT2-FLAG was incubated with cellular lysate with or without 1 mM NAD *in vitro*. Specified reactions were incubated with 5 mM nicotinamide or 50 μM sirtinol. One half of reaction products were visualized by Western blotting with specific antibody (up). The other half of the immunoprecipitated material was incubated with PEPCK1 antibody and protein A agarose then washed and visualized by Western blotting with antibody for pan-acetylated protein and PEPCK1 (down). (**D**) HEK293T cells were transfected with flag tagged wild type or mutant SIRT2 (H187Y) plasmid for 36 hours, then treated with indicated 5 mM NAM or 50 μM sirtinol for 4 hours. Cells were harvested and lysated. One half of lysates were visualized by Western blotting with specific antibody (up). The other half of the lysates were immunoprecipitated with flag-beads, then washed and visualized by Western blotting with antibody for pan-acetylated protein and PEPCK1 (down).

Since Lys70, Lys71, and Lys594 were identified as key acetylated sites of human PEPCK1 and these are deacetylated by SIRT2^3, 25^, we first synthesized a peptide containing two acetylated sites (Lys70, Lys71) of PEPCK1 and incubated it with prokaryotic purified SIRT2 protein. We then assessed peptides by mass spectrometry (MS) (Fig. 1A). As expected, the deacetylation effect of SIRT2 was abolished by sirtinol in dose-dependent manner. Next, using a eukaryotic purified SIRT2 deacetylated system (Fig. 1B), we found that Lys40-acetylation levels of tubulin and overall-acetylation levels of PEPCK1 were significantly reduced following SIRT2 and its coenzyme treatment. However, this effect was eliminated after treating with either NAM or sirtinol, resulting in hyperacetylation of tubulin and PEPCK1 (Fig. 1C). We further studied the inhibitory effect of sirtinol in HEK293T cells, and consistent with previous deacetylation results, the deacetylation effect of SIRT2 was abolished after treatment with either NAM or sirtinol (Fig. 1D). As His187 is the key conserved site controlling the activity of SIRT2, mutant SIRT2 or inhibitor treatment caused no significant change to the acetylation of target proteins compared to the wild type of SIRT2 (Fig. 1D). Taken together, these results suggest that sirtinol increases the acetylation of PEPCK1 by inhibiting the activity of SIRT2 specifically.

### Sirtinol increases the acetylation of PEPCK1 at three key acetylation sites

**Figure 2:**
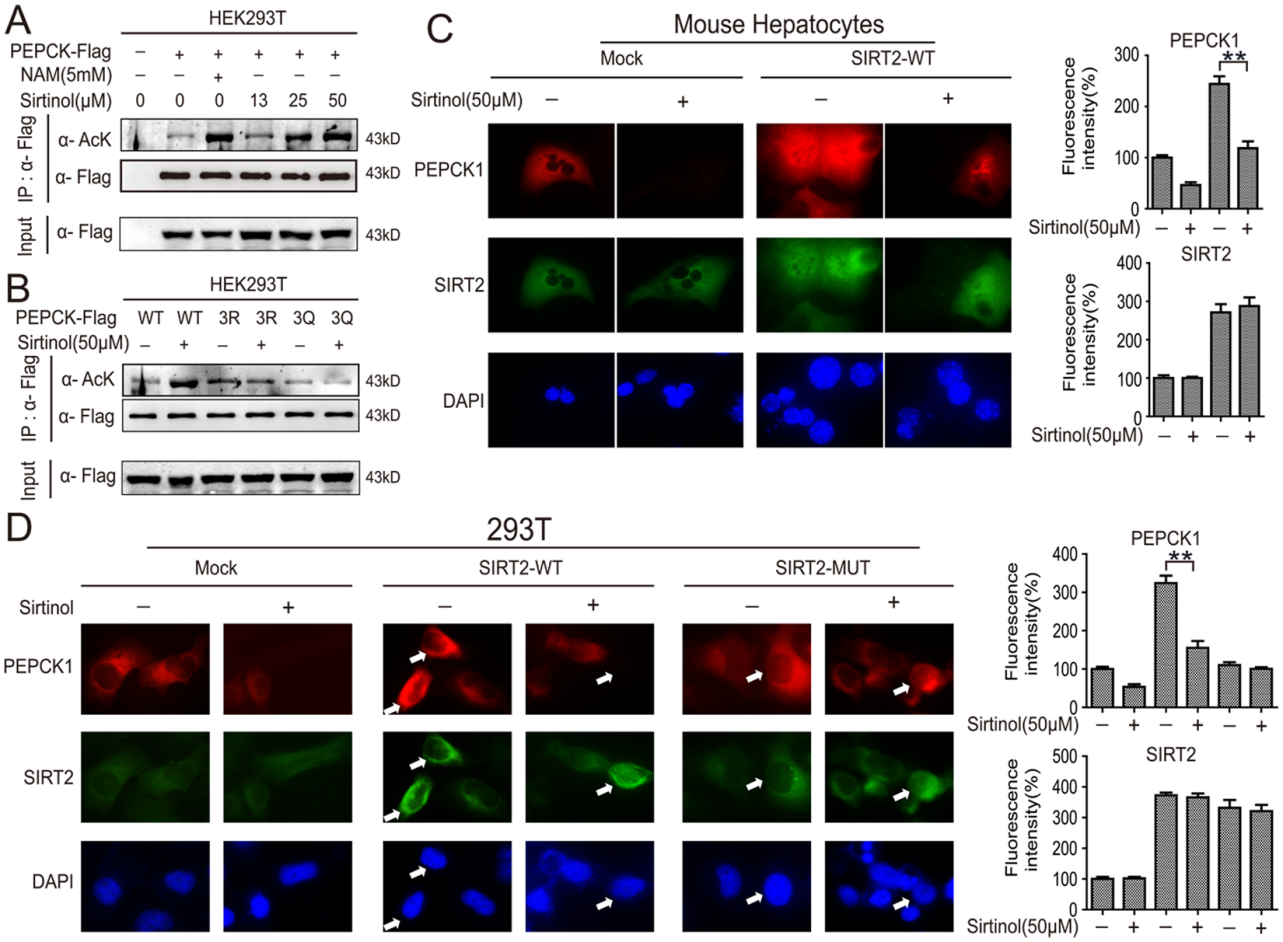
Sirtinol increases the acetylation of PEPCK1 at three key acetylation sites. (**A**) HEK293T cells were transfected with flag tagged PEPCK1 plasmid and then treated with indicated sirtinol for 4 hours. NAM was used as a positive control. Cells were harvested and lysated, then incubated with flag-beads. The immunoprecipitated protein from cell lysates were analyzed via Western blotting and FLAG are shown as loading controls. (**B**) HEK293T cells were transfected with flag tagged wild type or mutant PEPCK1 (3K/R and 3K/Q) plasmid, then treated with 50 μM sirtinol for 4 hours. Cells were harvested and lysated, then incubated with flag-beads, the immunoprecipitated protein from cell lysates were analyzed by Western blotting and FLAG are shown as loading controls. (**C**) Primary mice hepatocytes were transfected with flag tagged SIRT2 plasmid, followed by treatment with 50 μM sirtinol for 4 hours. PEPCK1 (red), SIRT2 (green) and nucleus (blue) were visualized by indirect immunofluorescence. (**D**) 293T cells were transfected with flag tagged SIRT2 plasmid, followed by treatment with 50 μM sirtinol for 4 hours. PEPCK1 (red), SIRT2 (green) and nucleus (blue) were visualized by indirect immunofluorescence.

Acetylation of three key acetylation sites of PEPCK1 (Lys70, Lys71 and Lys594) leads to destabilized proteins, accompanied by decreased levels of PEPCK1^25^. We further explored whether the increased overall-acetylation of PEPCK1 caused by sirtinol is associated with hyperacetylation of these key sites. HEK293T cells were overexpressed with flag-tagged *PEPCK1* then treated as indicated. Sirtinol increased the overall-acetylation levels of PEPCK1 significantly in dose-dependent manner (Fig. 2A). However, the hyperacetylation inducing effect of sirtinol was abolished after the lysines (K) of three acetylation sites of PEPCK1 mutated to arginines (R) or glutamine (Q), resulting in marginal acetylation change of PEPCK1 as compared to the untreated group (Fig. 2B). Hyperacetylation of three key acetylation sites of PEPCK1 resulted its decreased protein levels^25^. As expected, SIRT2 overexpressed hepatocytes had an increased PEPCK1 level as compared to hepatocytes, which transfected with control plasmid. This effect was attenuated following treatment with sirtinol (Fig. 2C and D). These results suggest that hyperacetylation caused by sirtinol is dominantly due to acetylation of Lys70, Lys71 and Lys594 of PEPCK1.

### Sirtinol decreases protein levels of PEPCK1 and cell gluconeogenesis

**Figure 3:**
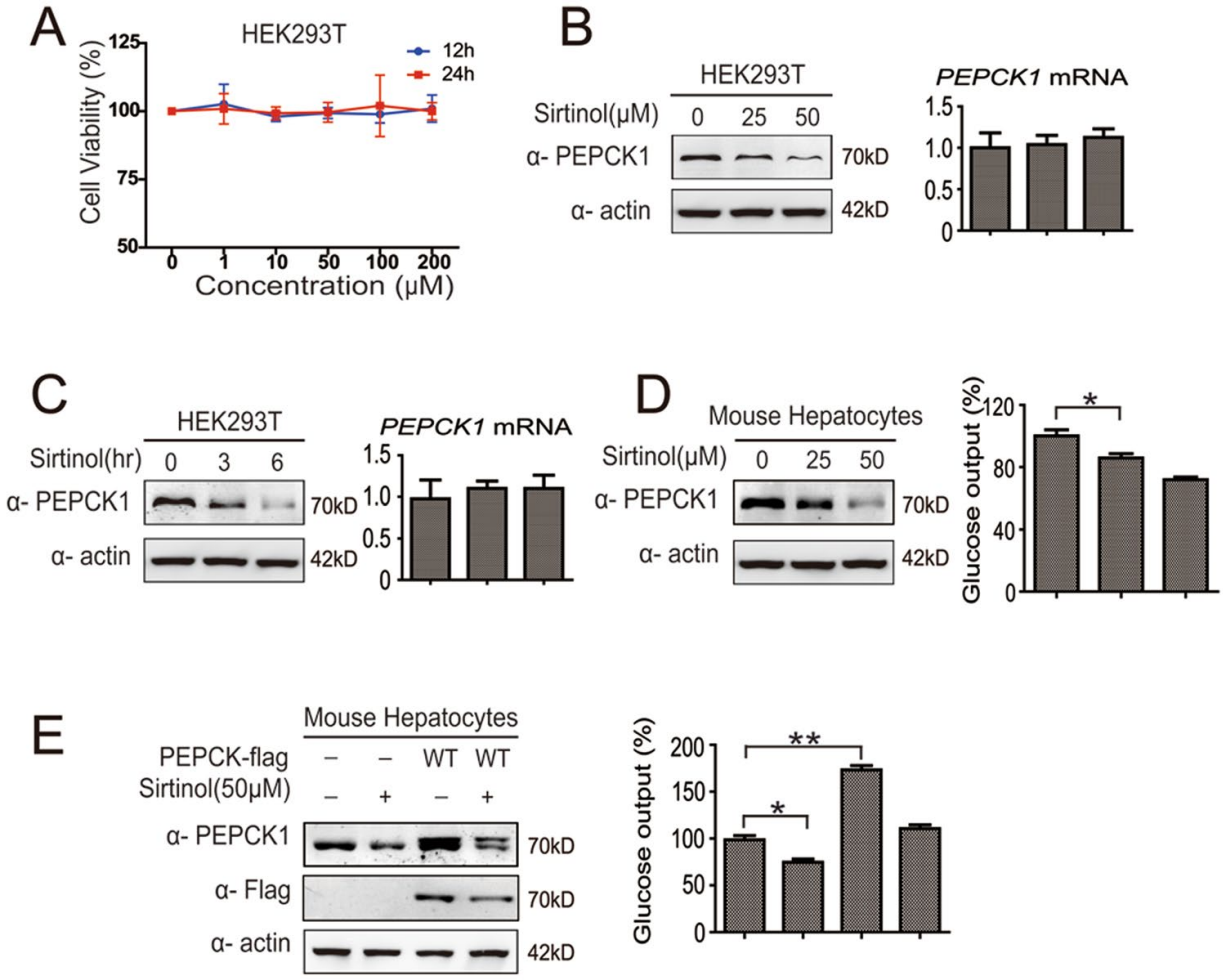
Sirtinol decreases protein levels of PEPCK1 and cell gluconeogenesis. (**A**) The cells were treated with indicated sirtinol (1–200 μM) for 12 and 24 hours. Cells viability was detected by CCK-8 assay. (**B**) HEK293T cells were treated with indicated sirtinol for 4 hours. Cells were harvested and visualized by Western blotting and RT-PCR. (**C**) HEK293T cells were treated with 50 μM sirtinol for indicated hours. Cells were harvested and visualized by Western blotting and RT-PCR. (**D**) Primary mice hepatocytes were treated with indicated sirtinol for 4 hours. Cells were then harvested and lysated, as well as visualized by Western blotting (left). Culture medium was collected and the glucose concentration was measured (right). (**E**) Primary mice hepatocytes were transfected with flag tagged PEPCK1 plasmid, as well as treated with 50 μM sirtinol for 4 hours. Cells were harvested and visualized by Western blotting (left). Culture medium was collected and the glucose concentration was measured (right). The data are represented as the means ± SEM from three independent experiments. *P < 0.05; **P < 0.01; NS, not significant.

Sirtinol exhibits an anti-proliferation effect to certain tumor cells^18–22^. Consequently, we assessed the cytotoxicity of sirtinol in HEK93T cells by the CCK-8 assay. HEK293T cells showed a tolerance to sirtinol even under as much as 200 μM sirtinol treatment (Fig. 3A). Using HEK293T and mouse hepatocytes, we found that sirtinol significantly reduced the PEPCK1 levels in a concentration- and time-dependent manner without changing its mRNA level (Fig. 3B, C and D). Since PEPCK1 plays an important role in gluconeogenesis and catalyzes its rate-limiting step^6–9^, we further explored cell gluconeogenesis capability. As expected, cell gluconeogenesis capability was closely related to protein levels of PEPCK1. Sirtinol significantly reduced the cell glucose output in a dose-dependent manner and gluconeogenesis decreased as much as 30% under 50 μM sirtinol treatment for 4 hours (Fig. 3D and E). Interestingly, sirtinol treatment did not degrade acetylation site mutated PEPCK1 proteins (SFig. A and B).

### Sirtinol-induced acetylation promotes PEPCK1 degradation via ubiquitin-proteasome pathway

**Figure 4:**
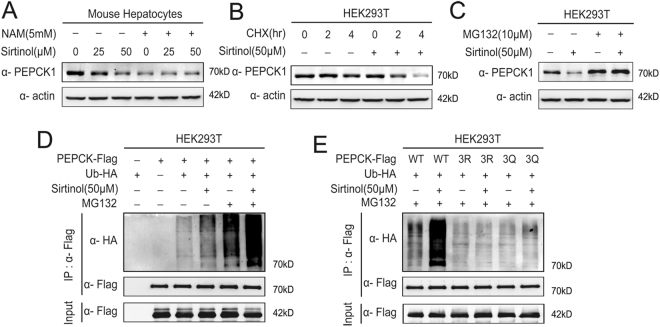
Sirtinol-induced acetylation promotes PEPCK1 degradation via ubiquitin-proteasome pathway. (**A**) Primary mice hepatocytes were treated with indicated sirtinol for 4 hours with or without 5 mM NAM for 6 hours. Cells were harvested and lysated, then visualized by Western blotting. (**B**) 293T cells treated with indicated CHX, with or without sirtinol 50 μM for 4 hours. Cells were harvested and lysated, and endogenous PEPCK1 was visualized by Western blotting. (**C**) 293T cells treated with indicated MG132 with or without sirtinol 50 μM for 4 hours. Cells were harvested and lysated, and endogenous PEPCK1 was visualized by Western blotting. (**D**) Flag-tagged PEPCK1 and HA-tagged ubiquitin were coexpressed in HEK293T cells, then treated with indicated sirtinol for 4 hours with or without 10 μM MG132 for 4 hours. Ubiquitination levels of affinity purified Flag-PEPCK1 proteins were detected and visualized by Western blotting. (**E**) Different phenotypic Flag-tagged PEPCK1 and HA-tagged ubiquitin were coexpressed in HEK293T cells, then treated with indicated sirtinol for 4 hours with or without 10 μM MG132 for 4 hours. Ubiquitination levels of affinity purified Flag-PEPCK1 proteins were detected and visualized by Western blotting.

In order to elucidate how sirtinol-induced hyperacetylation of PEPCK1 promotes PEPCK1 degradation, primary mouse hepatocytes were each treated with sirtuins deacetylase inhibitor nicotinamide (NAM), a chemical that supposedly inhibits all Class III deacetylases^26^. In confirmation with our previous finding^25^, levels of endogenous PEPCK1 were decreased significantly (Fig. 4A). Accordingly, the PEPCK1 decreasing effect of sirtinol can be abolished by the overdose of the deacetylase inhibitor (Fig. 4A). Next, we inhibited PEPCK1 synthesis by using CHX (Cycloheximide) and found that PEPCK1 degraded faster in presence of sirtinol (Fig. 4B). Furthermore, when MG132 was included to inhibit proteasomal degradation, sirtinol-induced PEPCK1 degradation was blocked (Fig. 4C). These results suggest that the ubiquitin-proteasome pathway mediates acetylation-promoted decrease of PEPCK1. Consistent with these results, active PEPCK1 ubiquitinylation was detected and sirtinol significantly increased PEPCK1 ubiquitinylation (Fig. 4D) after Flag-tagged PEPCK1 and HA-tagged ubiquitin were coexpressed in HEK293T cells,. However, the role of sirtinol as promoter of PEPCK1 ubiquitination was not observed after lysines (K) on three acetylation sites of PEPCK1 mutated to arginines (R) or glutamine (Q) (Fig. 4E).

### Sirtinol destabilizes PEPCK1 and inhibits gluconeogenesis by targeting on SIRT2

**Figure 5:**
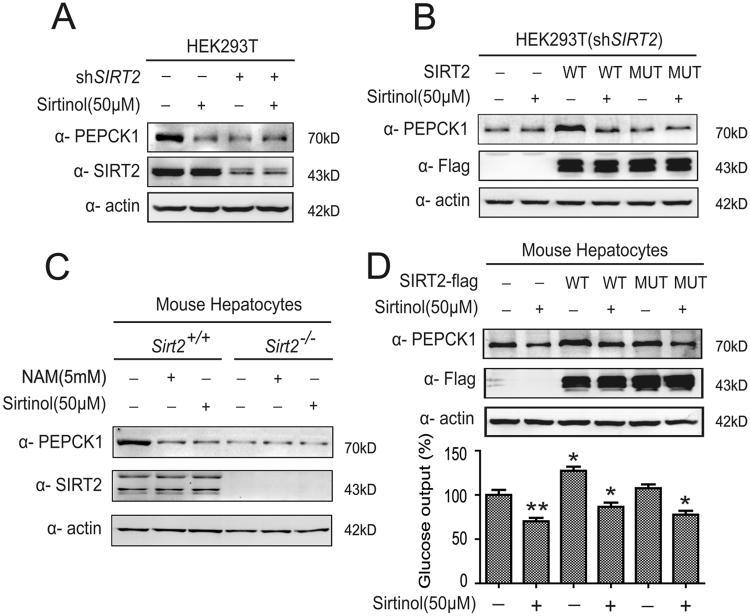
Sirt2 Deacetylates and Stabilizes PEPCK1. (**A**) HEK293T cells with Sirt2 knocked down were treated with indicated sirtinol for 4 hours. Sirt2 and endogenous PEPCK1 was visualized by Western blotting. (**B**) Different phenotypic Flag-tagged SIRT2 were transfected to HEK293T cells with Sirt2 knocked down, then treated with indicated sirtinol for 4 hours. Cells were harvested and lysated, then visualized by Western blotting. (**C**) Different phenotypic primary mice hepatocytes were cultured overnight, then treated indicated sirtinol for 4 hours or indicated NAM for 6 hours. Cells were harvested and lysated, then visualized by Western blotting. (**D**) Different phenotypic Flag-tagged SIRT2 were transfected to primary mice hepatocytes, then treated with indicated sirtinol for 4 hours. Cells were harvested and lysated, then visualized by Western blotting (up). Medium was collected and the glucose concentration was measured (down).

As a NAD^+^-dependent class III deacetylase, SIRT2 deacetylates and stabilizes PEPCK1^25^. We investigated whether SIRT2 is primarily responsible for decreasing protein levels of PEPCK1 and cell gluconeogenesis of sirtinol. We found that knockdown of *SIRT2* by shRNA significantly decreased endogenous PEPCK1 levels and blocked the PEPCK1 decreasing effect of sirtinol (Fig. 5A). Furthermore, when wild-type *SIRT2*, but not its catalytically inactive H187Y mutant^27, 28^, was put back in *SIRT2*-knockdown 293T cells, a significant increase of PEPCK1 protein levels was observed. Moreover, sirtinol reversed the PEPCK1 increasing effect of wild-type *SIRT2* (Fig. 5B). Hepatocytes from *Sirt2* ^−/−^ mice had a decreased endogenous PEPCK1 level compared to their counterparts, and *Sirt2* knock out also blocked the PEPCK1 decreasing effect of sirtinol (Fig. 5C), consistent with *SIRT2*-knockdown results from 293T cells. This validated our model that acetylation destabilizes PEPCK1 and that PEPCK1 protein levels are closely related to the amount of gluconeogenesis, which is regulated by sirtinol (Fig. 5D). In addition, since sirtinol has inhibitory effect on SIRT1, we further explored whether the inhibitor or agonist of SIRT1 could influence the protein levels of PEPCK1 and found that SIRT1 did not affect the protein levels of PEPCK1 (SFig. C).

### Sirtinol destabilizes PEPCK1 and inhibits gluconeogenesis in mice

**Figure 6:**
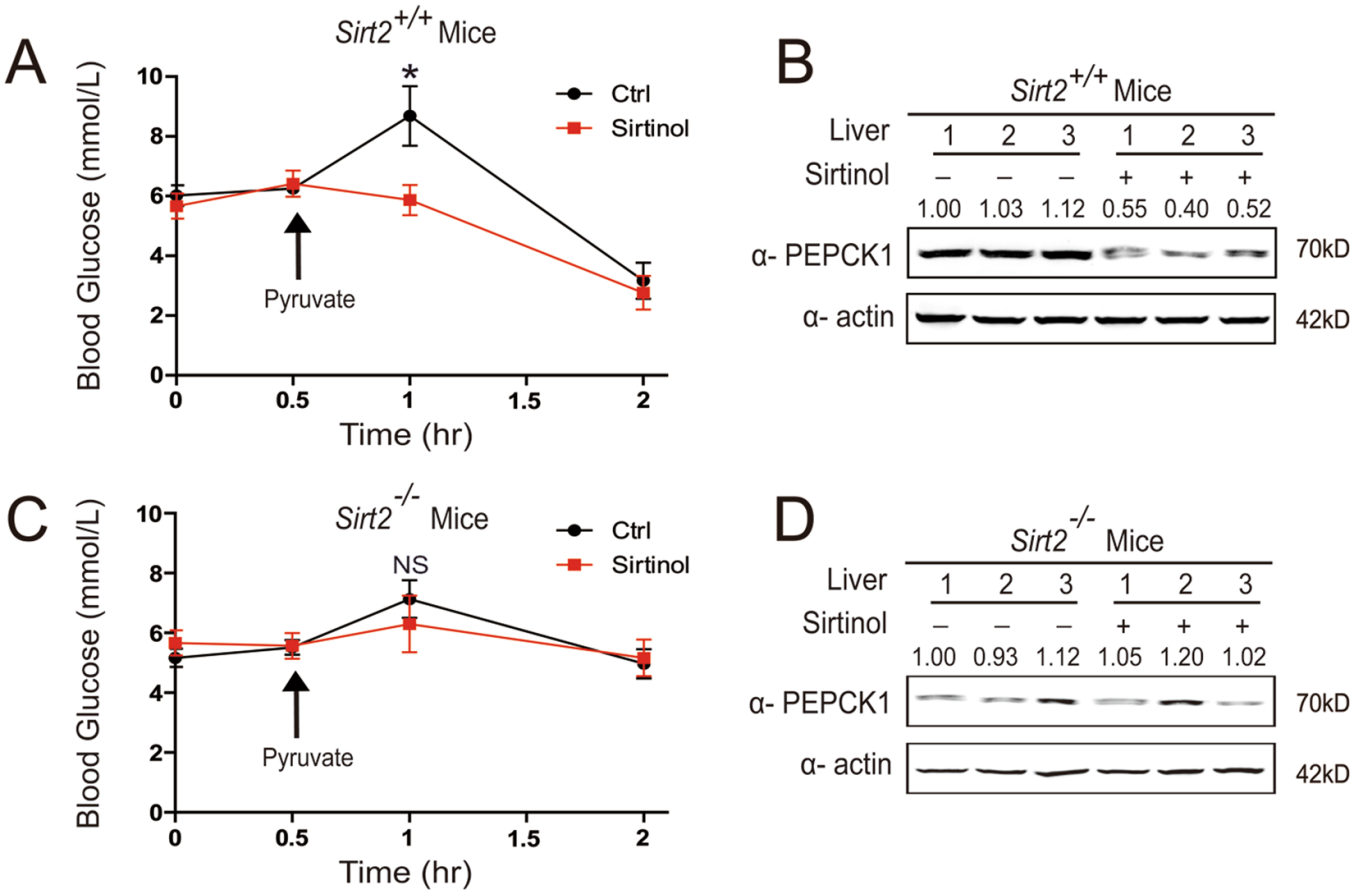
Sirtinol destabilizes PEPCK1 and inhibits gluconeogenesis in mice. (**A**) sirt2^+/+^ mice (n = 6) were starved overnight, then blood glucose were measured at different time points as indicated. Two groups of mice were injected with saline and sirtinol (10 mg/kg body weight) respectively at the begining. Then pyruvate (2 g/kg body weight) were injected as indicated. (**B**) sirt2^+/+^ mice were sacrificed after pyruvate tolerance tests, liver tissue were harvested and lysated, then visualized by Western blotting. (**C**) sirt2^−/−^ mice (n = 6) were starved for overnight, then blood glucose were measured at different time points as indicated. Two groups of mice were injected with saline and sirtinol (10 mg/kg body weight) respectively at the begining. Then pyruvate (2 g/kg body weight) were injected as indicated. (**D**) sirt2^−/−^ mice were sacrificed after pyruvate tolerance tests, liver tissue were harvested and lysated, then visualized by Western blotting. The data are represented as the means ± SEM. *P < 0.05; **P < 0.01; NS, not significant.

Finally, we investigated whether PEPCK1 level and gluconeogenic rate could be regulated by injecting sirtinol in mice. Since pyruvate is a source for gluconeogenesis, we employed pyruvate tolerance tests. At 1 hour after intraperitoneal injection, sirtinol effectively reduced blood glucose levels by approximately 40% compared to the control group in mice (Fig. 6A). Simultaneously, sirtinol decreased PEPCK1 protein levels in mice liver (Fig. 6B), and *Sirt2* ^−/−^ mice displayed low gluconeogenic ability. Furthermore, sirtinol could not decrease PEPCK1 or blood glucose (Fig. 6C and D). This was consistent with our *in vitro* findings. Together, these results demonstrate that sirtinol-induced PEPCK1 destabilization may serve as an important mechanism to regulate the rate of gluconeogenesis.

## Discussion

Biological anti-diabetic strategies have been widely developed in the past few decades. Nevertheless, new chemical anti-diabetic drugs are so few that the clinical outcome for advanced diabetic patients remains dependent on insulin injections. Growing evidence suggests that acetylation is a conservative protein modification and that regulation of glucose metabolism enzymes may affect gluconeogenesis. This provides a new strategy to develop anti-diabetic drugs by regulating protein acetylation^2, 3, 25^. In this study, we have uncovered that sirtinol, a SIRT2 inhibitor, destabilized the metabolic enzyme PEPCK1 and decreased gluconeogenesis *in vitro* and *in vivo*.

Sirtinol selectively inhibits NAD^+^-dependent deacetylases SIRT1 and SIRT2, as it shares the β-naphthol pharmacophore with other competitive sirtuin inhibitors, such as cambinol and splitomicin^29^. SIRT1, one of the most widely reported members of the SIRT family, is known to regulate cell proliferation, apoptosis, and migration^13, 30^. Targeting SIRT1, sirtinol-caused hyperacetylation of p53, Ku70, and FOXO3a, as well as phosphorylation of MAPK, leads to significant cytotoxic effect on breast and colon cancer cells^21, 22^. However, our cell viability test and mouse pyruvate tolerance test demonstrated that sirtinol was well tolerated in the absence of other noxious stimuli. These may be due to less malignant cells and in absence of stress stimuli (e.g. cell starvation, sirtinol overdose, and chemotherapeutic agents) in our study.

SIRT2, broadly conserved through organisms ranging from bacteria to humans, catalyzes a wide range of biological processes including genetic control, development, and metabolism, in which the removal of the acetyl group from lysine residues is coupled with the hydrolysis of NAD to generate nicotinamide, lysine, and O-acetyl-ADP-ribose^11, 14–16^. There are many SIRT2 inhibitors that are generally classified into different groups: nicotinamide, as a substance that inhibits NAD^+^-dependent reactions in general^31, 32^; and SIRT2-specific inhibitors, such as sirtinol^33^, spitomicin^34^ and cambinol^18^. A well-studied substrate of SIRT2 is α-tubulin. SIRT2 deacetylates lysine-40 of α-tubulin both *in vitro* and *in vivo*, and the acetylation of α-tubulin was used as an indicator of SIRT2 activity in our study^17^. PEPCK1 is another substrate of SIRT2. In both yeast and humans, acetylation of PEPCK1 has been reported, and its catalytic activity is inactivated following acetylation^3, 10^. Lys70, Lys71, and Lys594 of human PEPCK1 was found to be acetylated, and acetylation of these sites led to decreased protein stability, reduced protein levels, and declined gluconeogenesis without affecting mRNA levels^3^. Consistent with previous studies, we found that inhibition of the SIRT family increased acetylation of PEPCK1, as well as destabilized it by inhibiting SIRT2 activity. However, compared with SIRT1-mediated translational control that usually takes hours to occur, this SIRT2-mediated post-translational modification control on PEPCK1 by sirtinol only took minutes. In addition, we further explored whether the inhibitor or agonist of SIRT1 could influence of PEPCK1 and found SIRT1 showed a negative affect on PEPCK1. Thus, SIRT2 inhibition, not SIRT1, might serve as a dominant and more efficient way to regulate gluconeogenesis. We propose that sirtinol plays a critical role in coordinating the level of PEPCK1 by SIRT2 inhibition. Consistent with our previous studies, this data demonstrate that SIRT2 is the major enzyme responsible for PEPCK1 acetylation and is the downstream target of sirtinol. Therefore, PEPCK1 stability is controlled by a regulatory network, including the balance between acetylation and deacetylation in response to sirtinol. Employing mass spectrometry experiments and catalytic-mutated phenotypic SIRT2, we found sirtinol to be mainly responsible for the post-translational modification control of PEPCK1, and SIRT2 is the direct target of sirtinol in regulating PEPCK1 degradation.

PEPCK1, which reverses the reaction catalyzed by pyruvate kinase in glycolysis, is the critical enzyme for gluconeogenesis and is linked with hepatic glucose output^5^. When sirtinol is present, the acetylated PEPCK1 is ubiquitinylated and subsequently degraded by proteasomes. Therefore, gluconeogenesis is suppressed. As for the acetylation sites, mutation of three lysines to glutamines (an acetylation mimetic) should increase PEPCK1 ubiquitination but it decreased it to the same levels as mutation of the same residues to arginines (a deacetylation mimetic). We believed these lysines residues were specific signals for degradation, modifications of other amino acids could not be recognized by ubiquitin. Our data shows that glucose output and PEPCK1 protein levels, both of which are regulated by sirtinol, were closely correlated *in vitro*. More importantly, these cells demonstrated tolerance to sirtinol even under as much as 200 μM of sirtinol treatment, which implied that the drug can be used *in vivo*. Indeed, pyruvate tolerance tests demonstrate that mice can tolerate sirtinol, and sirtinol inhibits the conversion of pyruvate to glucose *in vivo*.

In the present study, we provide insight into the regulation of sirtinol on cellular gluconeogenesis. Sirtinol decreased liver PEPCK1 protein level and prevented pyruvate-induced blood glucose from increasing. Based on our results, rate-limiting enzyme PEPCK1 is the downstream target of sirtinol, and the inhibition of SIRT2 activity plays an important role in cell gluconeogenesis. Thus, SIRT2 may be a novel molecular target for diabetes therapy, and may shed light on the underlying diabetes treatment mechanism of sirtinol.

## Materials and Methods

### Ethics, consent and permissions

All experiments utilizing animals and cells were approved by the Ethical Committee of Medical Research of Fudan University. All animal experiments conformed to protocols approved by animal care and use committees at Fudan University.

### Cell culture and reagents

The HEK293T cell line was a gift from the Zhao lab of Fudan University (Shanghai, China). Cells were maintained in Dulbecco’s modified Eagle’s medium (DMEM) (Invitrogen, Carlsbad, CA, USA) containing 10% fetal bovine serum (Invitrogen), penicillin (Invitrogen) (100 U/ml), and streptomycin (Invitrogen) (100 U/ml). Full-length *PEPCK1* (wildtype, 3K/R and 3K/Q) and *SIRT2* (wildtype and H187Y) plasmids were also gifts from the Zhao lab of Fudan University (Shanghai, China). Plasmids were cloned to Flag- or HA-tagged destination vectors according to different needs. Point mutations for *PEPCK1* and *SIRT2* were generated by site-directed mutagenesis. Antibodies against Flag (Sigma, St. Louis., MO, USA), HA (Santa Cruz, Dallas, TX, USA), PEPCK1 (Santa Cruz), SIRT2 (Sigma), α-Tubulin (CST, Danvers, MA, USA), acetylated α-Tubulin (Abcam, Cambridge, UK), and β-actin (Sigma) were all purchased, and the polyclonal antibody against acetyl-lysine was a gift from the Zhao lab. Trichostatin A (CST), nicotinamide (Sigma), sirtinol (Selleck, Houston, TX, USA), MG132 (Sigma), EX527 (Selleck), SRT1720 (Selleck), and CHX (Sigma) were all purchased. Control and siSirt2 adenovirus were purchased (Vector Biolabs, Malvern, PA, USA).

### Primary hepatocytes

Primary hepatocytes were isolated from fed adult mice by a modified version of the collagenase method^35^. The cells were plated in M199 medium containing 10% fetal bovine serum (Invitrogen), penicillin (Invitrogen) (100 U/ml), streptomycin (Invitrogen) (100 U/ml), and 500 nM dexamethasone (dex; Sangon Biotech, Shanghai, China) at a density of 5 × 10^5^ cells/well on 6-well plates or 5 × 10^6^ cells/100-mm cell culture plate. After attachment (2 hours), the medium was removed and fresh medium was added for 16 hours, followed by drug treatment as described below.

### Western blot

Standard procedures were followed for western blot, except for the detection of acetylation, which used 50 mM Tris (pH 7.5) with 10% (v/v) Tween 20 and 1% peptone (AMRESCO, Solon, OH, USA) as a blocking buffer. Primary and secondary antibodies were diluted in 50 mM Tris (pH 7.5) with 0.1% peptone. Signals were probed using the chemiluminescence ECL plus reagent (Thermo, Grand Island, NY, USA) and detected using a Typhoon FLA9500 scanner (GE, Fairfield, CT, USA).

### Glucose output test

Primary hepatocytes changed with Krebs–Henseleit–HEPES buffer (1.2 mM MgSO_4_ (Sigma), 1.2 mM KH_2_PO_4_ (Sigma), 2.5 mM CaCl_2_ (Sangon), 4.7 mM KCl (Sangon), 25 mM NaHCO_3_ (Sigma), 25 mM HEPES (Sigma), 120 mM NaCl (Sangon), pH = 7.4) containing 20 mM sodium lactate (Sigma), and 2 mM sodium pyruvate (Sigma). At the end of the incubation period, medium glucose was quantified using glucose assays kit (GAGO20; Sigma), and normalized to the total protein content per well.

### Deacetylation assay

Cells were lysed in NP-40 buffer containing 50 mM Tris-HCl (Sigma), 150 mM NaCl (Sangon), 0.5% Nonidet P-40 (Sigma), 1 μg/ml aprotinin (Sigma), 1 μg/ml leupeptin (Sigma), 1 μg/ml pepstatin (Sigma), 1 mM Na_3_VO_4_ (Sigma), and 1 mM PMSF (Sigma), pH = 7.5. For immunoprecipitation, 500 μl of cell lysate was incubated with HA antibody for three hours at 4 °C with rotation. Then, 30 μl Protein A Agarose (Millipore, Billerica, MA, USA) was added for 12 hours at 4 °C with rotation, and the beads were washed three times with lysis buffer before proteins were dissolved in loading buffer. The SIRT2 assay was done using bacterial expression and purification (Biovision, Milpitas, CA, USA). Deacetylation assays were carried out in the presence of 5 μg enzyme and 0.3 μg peptide in 30 μl reaction buffer (30 mM HEPES (Sigma), 0.6 mM MgCl_2_ (Sangon), 1 mM DTT (Sigma), 1 mM NAD^+^ (Sigma), and 10 mM PMSF (Sigma)). The deacetylation reaction was incubated for 3–5 hours at 37 °C before the mixture was de-salted by passing it through a C18 ZipTip (Millipore). The de-salted samples were analyzed using a MALDI-TOF/TOF mass spectrometer (Applied Biosystems, Grand Island, NY, USA). The acetylated peptide used in the assay was GILRRLK^Ac^K^Ac^YDNCWL (Glssale, Shanghai, China).

### Ubiquitination Assay

Thirty-six hours following transfection, cells were lysed in 1% SDS buffer (Tris (Sigma), 0.5 mM EDTA (Sigma) and 1 mM DTT (Sigma), pH = 7.5), as well as boiled for 10 min. For immunoprecipitation, the lysates were diluted 10-fold in Tris-HCl buffer. Analyses of ubiquitination were performed using anti-HA blotting.

### Cell viability assay

Cells viability was determined using the CCK-8 colorimetric assay in 96-well plates (2 × 10^3^ cells/well) (Dojindo, Minato-ku, Tokyo, Japan). The absorbance at 450 nm was recorded using a micro-plate reader.

### Animal Experiments

Mice were purchased from the Department of Laboratory Animal Science, Fudan University. Mice were treated with sirtinol at a dose of 10 mg/kg bodyweight in 100 ml volume intraperitoneal injection twice a week for three weeks. At 1 hour following tail vein injection, blood was collected from the tail vein for glucose detection (Roche). Tissues were collected rapidly from anaesthetized mice and frozen in liquid nitrogen for further analysis.

### Statistics

Data was expressed as means ± standard error of the mean (SE). The data was analyzed through one-way ANOVAs followed by post hoc Duncan tests (SPSS 17.0). P < 0.05 was considered significant.

## Electronic supplementry meterial

Supplementary Info File
