# Supplementary material for: Sirtinol promotes PEPCK1 degradation and inhibits gluconeogenesis by inhibiting deacetylase SIRT2
Source: Sci Rep. 2017 Feb 28;7:7. doi: 10.1038/s41598-017-00035-9 (PMC5428341; doi:10.1038/s41598-017-00035-9)
Supplement: Supplementary file 1 — Supplementary Info File [file 41598_2017_35_MOESM1_ESM.pdf]

**Sirtinol promotes PEPCK1 degradation and inhibits gluconeogenesis by inhibiting deacetylase SIRT2**

Mingming Zhang[1,2,#], Yida Pan[2,#], Robert G Dorfman[3], Yuyao Yin[1], Qian Zhou[4], Shan Huang[5], Jie Liu[2,*], Shimin Zhao[4,*]

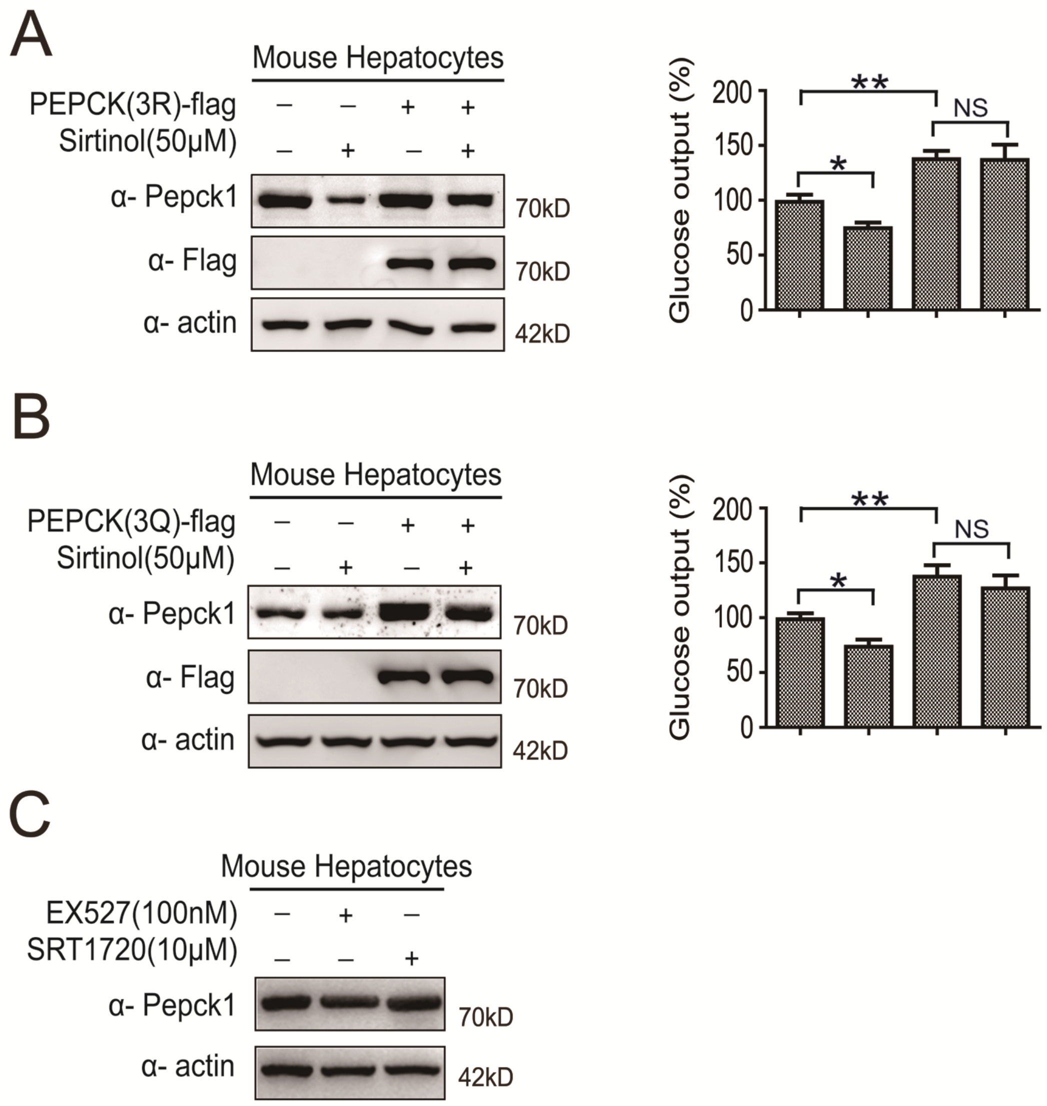


Supplementary Figure.

(A) Primary mice hepatocytes were transfected with flag tagged mutant PEPCK1(3K/R) plasmid, as well as treated with 50 μM sirtinol for 4 hours. Cells were harvested and visualized by Western blotting (left). Culture medium was collected and the glucose concentration was measured (right).

(B) Primary mice hepatocytes were transfected with flag tagged mutant PEPCK1(3K/Q) plasmid, as well as treated with 50 μM sirtinol for 4 hours. Cells were harvested and visualized by Western blotting (left). Culture medium was collected and the glucose concentration was measured (right).

(C) Primary mice hepatocytes were treated with 100nM EX527 or 10μM SRT1720 for 4 hours. Cells were harvested and visualized by Western blotting.
